# Prenatal Exposure to BPA: The Effects on Hepatic Lipid Metabolism in Male and Female Rat Fetuses

**DOI:** 10.3390/nu13061970

**Published:** 2021-06-08

**Authors:** Claudia Tonini, Marco Segatto, Simona Bertoli, Alessandro Leone, Arianna Mazzoli, Luisa Cigliano, Laura Barberio, Maurizio Mandalà, Valentina Pallottini

**Affiliations:** 1 Department of Science, University Roma Tre, Viale Marconi 446, 00146 Rome, Italy; claudia.tonini@uniroma3.it; 2 Department of Biosciences and Territory, University of Molise, Contrada Fonte Lappone, 86090 Pesche, Italy; marco.segatto@unimol.it; 3 International Center for the Assessment of Nutritional Status (ICANS), Department of Food Environmental and Nutritional Sciences (DeFENS), University of Milan, Via Mangiagalli 25, 20133 Milan, Italy; simona.bertoli@unimi.it (S.B.); alessandro.leone1@unimi.it (A.L.); 4 Lab of Nutrition and Obesity Research, Istituto Auxologico Italiano, IRCCS, 20100 Milan, Italy; 5 Department of Biology, University of Naples Federico II, Complesso Universitario Monte Sant’Angelo, Via Cinthia—Edificio 7, 80126 Naples, Italy; arianna.mazzoli@unina.it (A.M.); luisa.cigliano@unina.it (L.C.); 6 Department of Biology, Ecology and Earth Science, University of Calabria, Arcavacata di Rende, 87036 Cosenza, Italy; laura.barberio90@gmail.com (L.B.); m.mandala@unical.it (M.M.); 7 Neuroendocrinology Metabolism and Neuropharmacology Unit, IRCSS Fondazione Santa Lucia, Via del Fosso Fiorano 64, 00143 Rome, Italy

**Keywords:** acyl coenzyme A carboxylase, bisphenol A, cholesterol, fatty acids, 3-hydroxy 3-methylglutaryl coenzyme A reductase, fetuses, liver

## Abstract

Bisphenol A (BPA) is an organic chemical compound widely used for manufacturing plastics. BPA exposure originates principally from the diet, but it can also originate from dermal contact. In over 90% of individuals, including pregnant women, BPA is detectable in several body fluids. The effects of this exposure on the fetus are under active investigation in several research laboratories. The aim of our work was to study the impact of prenatal exposure to BPA in the liver of rat fetuses from a sex-dependent point of view. We particularly investigated the effects of prenatal BPA exposure on hepatic lipids because of their crucial role, not only for the liver, but also for the whole-body functions. Our results demonstrate that the liver of rat fetuses, in utero exposed to a very low dose of BPA (2.5 µg/kg/day), displays significant modulations with regard to proteins involved in cholesterol and fatty acid biosynthesis and trafficking. Moreover, an impact on inflammatory process has been observed. All these effects are dependent on sex, being observable only in female rat fetuses. In conclusion, this work demonstrates that maternal exposure to BPA compromises hepatic lipid metabolism in female offspring, and it also reveals the perspective impact of BPA on human health at doses currently considered safe.

## 1. Introduction

Bisphenol A (4,4-isopropylidenediphenol, BPA) is a plasticizer material particularly adapted for the industrial production of phenol and epoxy resins and polycarbonate plastics. Because of its high resistance to a broad range of temperatures and acids, it is abundantly used in manufacturing commonly used items [1], making BPA exposure unavoidable in daily life. Indeed, over 90% of individuals have a detectable amount of BPA in their body fluids [2]. BPA exposure occurs mainly through environmental pollution, inhalation, ingestion, or dermal contact. The most important route of intake is diet, as under certain physical or chemical conditions, the BPA present in the containers used for the storage of food and drinks can be released [3]. After ingestion, BPA is promptly metabolized and produces inactive metabolites, such as BPA-glucuronide, BPA-sulfate, and is finally excreted primarily through feces (56–82%) [4]. Nevertheless, levels of free BPA have been measured in the urine of adults and children [5], in the serum of pregnant women [6], as well as in amniotic fluid, umbilical cord blood [7], and breast milk [8]. Concerns have arisen about the presence of BPA in maternal and fetal fluids, which results in BPA exposure during pre- and perinatal periods, with long-term hazardous consequences on the offspring’s development and health [9,10]. Growing epidemiological studies reveal a correlation between urinary levels of BPA and increased risk for onset of metabolic disorders, e.g., type 2 diabetes, obesity, metabolic syndrome, and cardiovascular disease [11,12]. Moreover, studies carried out on animals have reported several disturbances in the metabolic profile due to exposure to low doses of BPA, including increased liver fat [13] and enhanced serum triglyceride and cholesterol levels in rodents [14]. In addition, early exposure to BPA has been shown to impair glucose metabolism and alters metabolic gene expression in male mice [15]. Notably, modified lipid metabolism has been observed not only in the liver and plasma of experimental models but also in the brain. In particular, it has been demonstrated that maternal exposure to low-dose BPA, via drinking water, affects cholesterol metabolism and critical signaling pathways in the brain of rat fetuses [16].

The liver is the key organ for the maintenance of lipid homeostasis. 3-Hydroxy-3-methylglutaryl-CoA reductase (HMGCR) and acetyl-CoA carboxylase (ACC) are the key and rate-limiting enzymes for cholesterol and fatty acid synthesis, respectively [17,18]. Lipid metabolism is primary regulated by a family of transcriptional factors, named sterol regulatory element binding proteins (SREBPs). Notably, SREBP1 is principally committed to the transcription of genes encoding enzymes involved in fatty acid metabolism, while SREBP2 mainly regulates the proteins controlling cholesterol metabolism [19]. Briefly, a low intracellular content of sterols induces the translocation of SREBP precursors from the endoplasmatic reticulum (ER) to the Golgi body, where they are proteolytically cleaved and form the active NH 2-terminal fragment (nSREBP). nSREBP enters the nucleus and induces the transcription of *acc* and *hmgcr* genes [20], as well as genes of receptors involved in lipid transport into and out of the liver: low-density lipoprotein receptor (LDLR) and scavenger receptor class B type 1 (SR-B1) [21,22].

Several studies have reported that the harmful effects of BPA on human health also rely on the exacerbated activation of inflammatory response [23,24], and the upregulation of pro-inflammatory cytokines, including interleukin-6 (IL-6) and tumor necrosis factor- α (TNF-α) [25]. Importantly, inflammation is the trigger for many chronic diseases, including diabetes, dyslipidemia, and cardiovascular disease [26,27]. It is accepted that gestational exposure can induce permanent modification in cells, organs, and tissues, without manifestation of symptoms until later in life. A plethora of studies has investigated consequences of early exposure to BPA in the post-natal period, while, to our knowledge, the possible presence of changes during fetal life remains poorly investigated. Thus, this work aims at studying the effects of maternal exposition to BPA on hepatic lipid metabolism in male and female fetuses.

## 2. Materials and Methods

### 2.1. Chemicals

**Figure 1:**
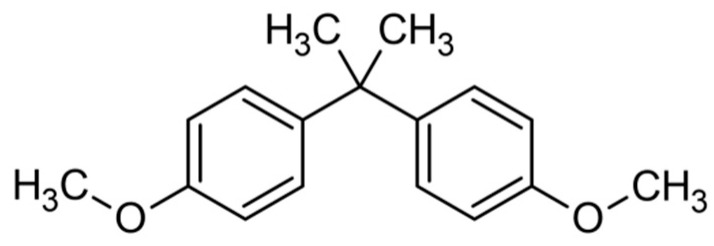
Chemical formula of bisphenol A (BPA).

BPA is an organic compound characterized by two hydroxyphenyl groups linked by a methyl bridge, belonging to the class of phenols (Figure 1). BPA (≥98% purity) used in this work was purchased from Sigma Aldrich (Milan, Italy).

### 2.2. Animals and Treatment

Experimental procedures were conducted in accordance with the European Guidelines for the care and use of laboratory animals (Directive 26/2014/EU) and were approved by the local ethical committee of the University of Calabria and by the Italian Ministry of Health (license n.74/2018-PR).

Female Sprague Dawley rats 8 weeks old were used. Animals were housed individually in the animal care facility and maintained in a regular light cycle (12 h light/dark photoperiod) and at 20–22 °C room temperature; food and water were *ad libitum* provided. Rats (six animals for group) were administered BPA (2.5 µg/kg/day) or its vehicle, ethanol 0.05% (Control, CTR) in drinking water, starting from 30 days before coitus and continued until gestational day (GD) 20. The actual intake was determined on the basis of the daily difference of drinking water volume.

A female in proestrus and a fertile male were placed overnight, and the following morning a vaginal smear was performed to detect spermatozoa, which confirms the first day of pregnancy (GD1). Animals were euthanized on GD 20 with isoflurane inhalation. Blood was collected to prepare the serum, and the uterus was removed. Fetus dissection and sex assessment were performed as previously described [16]. Liver tissues collected from each fetus were stored at −80 °C.

### 2.3. Measurement of Triglycerides and Cholesterol, HDL, and LDL Content in Serum and Liver Samples

The measurements of triglycerides and total, LDL, and HDL cholesterol in serum samples of pregnant rats were performed with an enzymatic method (Cobas Integra 400 Plus, Roche Diagnostics, Rotkreuz, Switzerland), with intra- and inter-assay coefficients of variation <2%. The triglyceride and cholesterol amount in fetal liver tissue were extracted and measured by the Triglyceride Quantification kit-MAK266 (Sigma-Aldrich, Milan, Italy) and Cholesterol Quantitation Kit-MAK043 (Sigma Aldrich, Milan, Italy), respectively, according to the manufacturer’s instructions. Briefly, for cholesterol measurement, lipids were extracted from tissue (10 mg) with 200 µL of chloroform:isopropanol: Nonylphenyl-polyethylene glycol solution (Nonidet P-40)(7:11:0.1), while triglycerides were solubilized from tissue (100 mg) using 1 mL of 5% Nonidet P-40 solution. Cholesterol esterase or lipase enzymes were added according to the manufacturer’s instruction in 50 μL of sample as the final experimental volume. In the presence of cholesterol esterase, total cholesterol, both free cholesterol and cholesteryl esters, was determined by enzymatic assay that resulted in a colorimetric product. Similarly, adding lipase enzyme, triglycerides were transformed to free fatty acids and glycerol, which, when oxidized, generate a colorimetric compound. The amount of cholesterol or triglycerides present in the samples was revealed by determining the absorbance at 570 nm, with Tecan Spark microplate reader (Männedorf, Switzerland). All samples were run in duplicate.

### 2.4. Measurement of Tumor Necrosis Factor Alpha (TNF-α) in Liver Samples

Slices of liver samples from rat fetuses were homogenized in homogenization buffer (Sucrose 0.1 M, potassium chloride (KCl) 0.05 M, potassium dihydrogen phosphate (KH2PO4) 0.04 M, ethylenediaminetetraacetic acid (EDTA) 0.04 M, pH 7.4). TNF-α concentration was evaluated by enzyme-linked immunosorbent assay (sandwich ELISA-assay), using the TNF-α Duo-Set kit (R&D, DBA, Milan, Italy), diluting each homogenate 1:50 [28] and revealed by Multiskan FC Microplate Photometer (Thermo Scientific, Monza, Italy). Data are expressed as ng of TNF-α per mg of proteins.

### 2.5. Total Lysate and Membrane Preparation for Western Blot Analysis

Fetal hepatic tissue was homogenized and sonicated in 1:10 *w/v* buffer containing sucrose 0.1 M, KCl 0.05 M, KH2PO4 0.04 M, EDTA 0.04 M, pH 7.4, plus 1:1000 protease inhibitor cocktail and 1:400 phosphatase inhibitor cocktail, Sigma-Aldrich, Milan, Italy using VCX 130 PB (Sonics, Newtown, CT 06,470, USA) and centrifuged at 12,200× *g* for 10 min at 4 °C to yield total lysate. Membrane fractions were isolated by centrifuging the total lysates at 24,000× *g* for 1 h at 4°C. Pellets were collected and homogenated by sonication. Proteins were quantified using the method from Lowry [29]. Laemmli buffer was used to dilute aliquots of homogenate samples, boiled for 5 min, and subjected to Western blot analysis.

### 2.6. Immunoblotting

Equal amounts of proteins (30 µg) from samples were separated by sodium dodecyl sulfate polyacrylamide denaturing gel electrophoresis (SDS-PAGE) (BioRad, Milan, Italy), using 7% or 13.5% polyacrylamide gels depending on the mw of the tested protein, as already described [16]. The following primary antibodies were tested: HMGCR (Abcam, ab242315, dilution 1:1000), ACC (Sigma-Aldrich, Milan, Italy SAB4501396, dilution 1:500), SR-B1 (Abcam, Cambridge, UK, ab52629, dilution 1:2000), LDLR (Abcam, Cambridge, UK, ab30532, dilution 1:1000), LRP1 (H80) (Santa Cruz Biotechnology, Santa Cruz, CA, USA, sc-25469, dilution 1:1000), ABC1 (AB.H10) (Santa Cruz Biotechnology, sc-58219, dilution 1:1000), SREBP2 (Abcam, Cambridge, UK, ab30682, dilution 1:1000), SREBP1 (Santa Cruz Biotechnology, Santa Cruz, CA, USA, sc-8984, dilution 1:1000), RhoA (Santa Cruz Biotechnology, Santa Cruz, CA, USA, sc-418, dilution 1:500), HRas (Santa Cruz Biotechnology, sc-53959, Santa Cruz, CA, USA, dilution 1:500), ERα (D12) (Santa Cruz Biotechnology, Santa Cruz, CA, USA, sc-8005, dilution 1:1000); p-NFκB p65 Ser536 (Santa Cruz Biotechnology, Santa Cruz, CA, USA, sc-33020, dilution 1:1000), NFκB p65 (Santa Cruz Biotechnology, Santa Cruz, CA, USA, sc-372, 1:3000), p-Stat3 (Santa Cruz Biotechnology, Santa Cruz, CA, USA, sc-8059, 1:1000), and Stat3 (Santa Cruz Biotechnology, Santa Cruz, CA, USA, sc-482, 1:3000). Antibodies against tubulin or vinculin (Sigma Aldrich, Milan, Italy, dilution 1:10000), or caveolin-1 (N20) (Santa Cruz Biotechnology, Santa Cruz, CA, USA, sc-894, dilution 1:3000), were used as loading control. Different housekeeping proteins were used depending on the molecular weight of the analyzed protein, in order to avoid confounding signals when detecting the immunoreactivity using the ChemiDoc MP system., BioRad, Milan, Italy Western blot images were analyzed as already described [16].

### 2.7. In Vitro HMGCR Degradation Assay

Liver samples (10 mg) were suspended in 100 µL TrisHCl 0.01 M (pH 7.4), sucrose 0.01 M, and then incubated at 37 °C for 0, 4, 8, 16, and 24 h. About 30 µg of protein was used for each reaction. Protein concentration was measured using the method of Lowry [29]. At the end of incubation, the reaction was blocked by adding a proper volume of Laemmli buffer. Samples were then analyzed through immunoblotting, as described in Section 2.6.

### 2.8. Statistical Analysis

Statistical analysis was performed by using unpaired Student’s *t* test, or by one-way or two-way analysis of variance (ANOVA), followed by Turkey post-hoc test, as specifically defined in the corresponding figure legends, and using six animals per experimental group. Data are displayed as mean ± standard deviation (SD), and *p* < 0.05 was considered significant. Statistical analysis and graphical illustrations were performed with GraphPad Prism v8.0 (GraphPad, La Jolla, CA, USA) for Windows.

## 3. Results

Pregnant rats were treated with 2.5 µg/kg/day BPA. We chose this very low dose on the basis of previous experiments carried on in our laboratories [16], demonstrating that, despite the current tolerable daily intake (TDI) indicated by the European Food Safety Authority (EFSA) is 4 μg/kg [30], a markedly lower dose exerts deleterious effects.

First, we assessed whether the exposition to 2.5 µg/kg/day of BPA affected parameters of lipid metabolism in pregnant rats, as it could affect fetal development. As shown in Table 1 there were no significant differences in serum levels of total cholesterol (T-C), triglycerides (TG), low-density lipoprotein-cholesterol (LDL-C) and high-density lipoprotein-cholesterol (HDL-C) in BPA-exposed animals compared to animals receiving vehicle (CTR). Moreover, no differences in serum BPA content were detected between exposed and not exposed dams.

**Figure 2:**
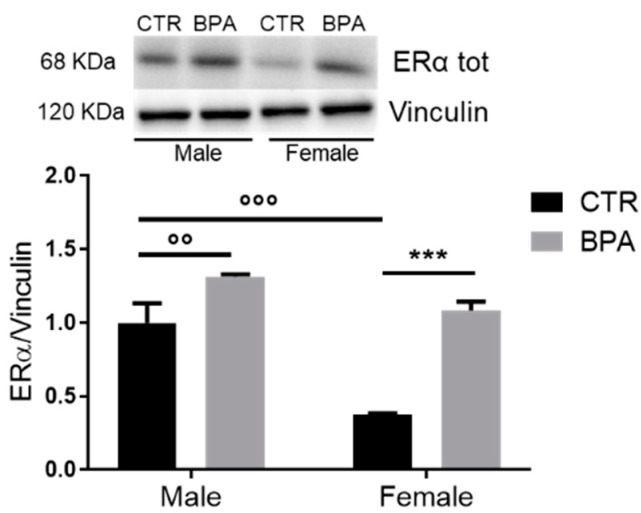
Effects of prenatal exposition to 2.5 µg/kg/day of BPA on ERα levels in fetal livers of male and female rats. Typical Western blot and densitometric analysis of ERα total levels. Vinculin was used to normalize protein loading. Values are expressed as mean ± SD, *n* = 6 for each experimental group. Differences between groups were considered as significant at *p* < 0.05 and were analyzed with two-way ANOVA, followed by Tukey post hoc test. °° *p* < 0.01 vs. CTR male; °°° *p* < 0.001 vs. CTR male; *** *p* < 0.001 vs. CTR female.

BPA is a widely recognized endocrine disruptor chemical (EDC) that may interact with many hormone receptors, thus disturbing their physiological function and increasing the risk of developing numerous diseases, including metabolic ones [9,10]. Thus, since we did not detect significant differences in serum BPA content, to ascertain whether BPA reaches the fetuses, we analyzed estrogen receptor α (ERα), whose protein levels tend to increase upon BPA stimulation [31]. As expected, the level of ERα significantly increased in fetuses prenatally exposed to BPA when compared to controls. The enhancement of ERα was observed in both sexes, with a greater effect in females (Figure 2). Interestingly, the physiological basal level observed in control fetuses was different between sexes, while BPA exposure makes ERα levels similar in female and male fetuses.

**Figure 3:**
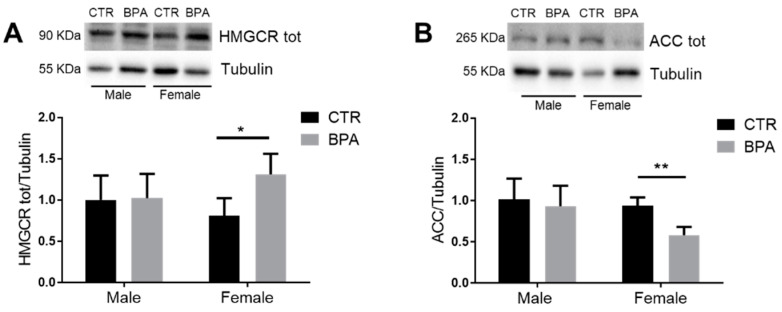
Effects of prenatal exposition to 2.5 µg/kg/day of BPA on HMGCR and ACC total levels in fetal livers of male and female rats. Typical Western blot and densitometric analysis of total HMGCR (**A**) and total ACC (**B**) levels. Tubulin was used to normalize protein loading. Values are expressed as mean ± SD, *n* = 6 for each experimental group. Differences between groups were considered as significant at *p* < 0.05 and were analyzed with two-way ANOVA, followed by Tukey post hoc test. * *p* < 0.05; ** *p* < 0.01 vs. CTR female.

To uncover the impact of maternal BPA exposure on fetal lipid metabolism, we analyzed the levels of key proteins involved in lipid biosynthesis and transport. First, we checked for the rate-limiting enzymes of cholesterol and fatty acid biosynthesis, namely HMGCR and ACC, respectively. The results showed that the prenatal exposure to BPA had a sex-dependent effect. Specifically, the levels of HMGCR protein significantly increased (Figure 3A), while ACC protein levels significantly decreased (Figure 3B) in BPA-exposed female fetuses compared to controls.

**Figure 4:**
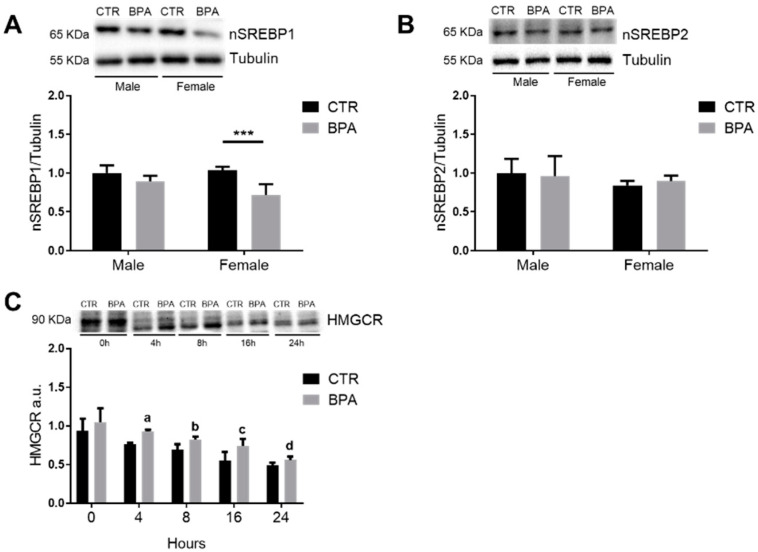
Effects of prenatal exposition to 2.5 µg/kg/day of BPA on SREBPs activation and HMGCR degradation rate in fetal livers of male and female rats. The figure shows the typical Western blot and the densitometric analysis of total HMGCR (**A**) and total ACC (**B**). Tubulin was used to normalize protein loading. Values are expressed as mean ± SD, *n* = 6 for each experimental group. Differences were considered as significant at *p* < 0.05 and were analyzed with two-way ANOVA, followed by Tukey post hoc test. *** = *p* < 0.001 vs. CTR female. (**C**) Representative Western blot and densitometric analysis of in vitro HMGCR degradation assay in female fetuses (for details see the main text). Data were analyzed with unpaired Student’s *t* test, and differences between each time point were considered as significant at *p* < 0.05. a = *p* < 0.001 vs. CTR 4 h; b = *p* < 0.5 vs. CTR 8 h; c = *p* < 0.5 vs. CTR 16 h; d = *p* < 0.5 vs. CTR 24 h.

In agreement with the reduced ACC protein expression observed in BPA-exposed female fetuses, the level of the nuclear and transcriptionally active fragment of SREBP1 (nSREBP1) was decreased in females, while no effects were observed in male fetuses (Figure 4A). On the contrary, there was no significant modulation in nSREBP2 protein levels, either in male or female fetuses prenatally exposed to BPA compared to control (Figure 4B). The increased HMGCR levels observed in female fetuses could depend on a reduced degradation rate of the enzyme following exposure to BPA, as shown in Figure 4C.

**Figure 5:**
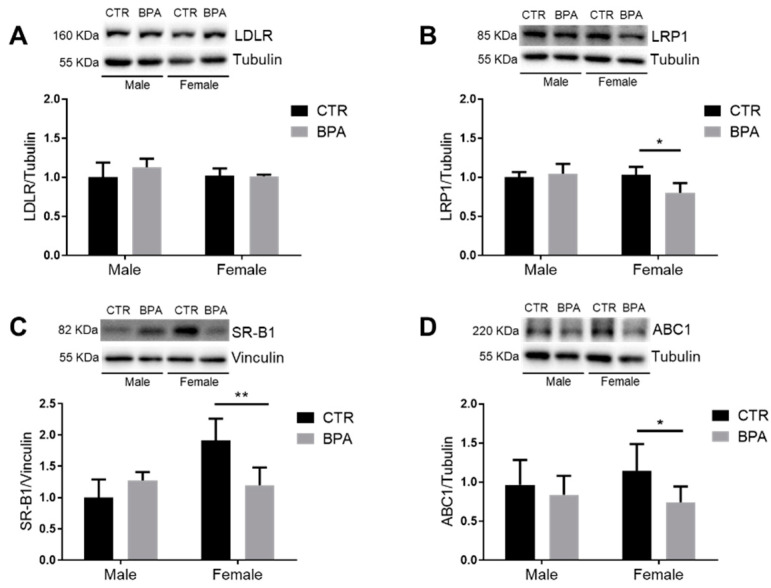
Effects of prenatal exposition to 2.5 µg/kg/day of BPA on cellular lipid import and export in fetal livers of male and female rats. The figure represents a typical Western blot and densitometric analysis of LDLR (**A**), LRP1 (**B**), SR-B1 (**C**), and ABC1 (**D**). Tubulin and vinculin were used to normalize protein loading. Values are expressed as mean ± SD, *n* = 6 for each experimental group. Differences between groups were considered as significant at *p* < 0.05 and were analyzed with two-way ANOVA, followed by Tukey post hoc test. * *p* < 0.5; ** *p* < 0.01 vs. CTR female.

Lipid homeostasis also relies on a series of lipoprotein membrane receptors and transporters [32]. In this study, we analyzed the effects of prenatal exposure to BPA on LDLR and Low density lipoprotein receptor-related protein 1 (LRP1), which are two main receptors involved in LDL-C uptake [33,34]. In addition, the scavenger receptor, class B type 1 (SR-B1) involved in the uptake of cholesteryl esters from HDL-C [35], and ATP-binding cassette transporter 1 (ABC1) which mediates the efflux of cholesterol and other lipids [36,37], were also assessed. The results showed that LDLR levels were not affected by BPA in male nor in female fetuses (Figure 5A). Conversely, the amount of LRP1, SR-B1, and ABC1 significantly decreased only in female fetuses exposed to BPA when compared to controls (Figure 5B–D).

As the previous results showed a sex-dependent modulation of proteins involved in lipid metabolism, the following experiments were performed only in female fetuses. Analyzing the main end-products of HMGCR and ACC, we found that the cholesterol and triglycerides contents in the liver of rat fetuses were not changed in fetuses exposed to BPA compared to controls (Table 2). Notably, the measurement of total and free cholesterol was similar, indicating that almost all cholesterol was present as free cholesterol, whereas cholesteryl esters were virtually absent in fetal hepatic tissue.

**Figure 6:**
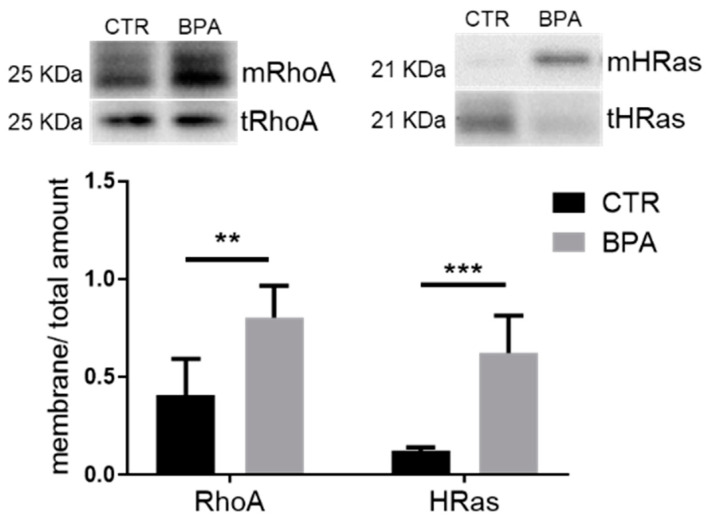
Effects of prenatal exposition to 2.5 µg/kg/day of BPA on RhoA and HRas prenylation in fetal livers of female rats. The figure shows typical Western blot of membrane-bound RhoA (mRhoA) and HRas (mHRas), and their total content in the lysate of female fetus livers from CTR animals and the ones prenatally exposed to BPA. The graph represents the ratio of membrane-bound and total levels of the proteins in liver lysates. Values are expressed as mean ± SD, *n* = 6 for each experimental group. Differences between groups were considered as significant at *p* < 0.05 and were analyzed with unpaired Student’s *t* test. ** *p* < 0.01; *** *p* < 0,001 vs. CTR.

Besides cholesterol, HMGCR is also responsible to produce isoprenoid intermediates, which regulate the subcellular localization and activation of small G-proteins [38]. In this work, the amounts of activated RhoA and HRas were measured in membrane lysates and considered as prototypes of geranyl-geranylated and farnesylated proteins, respectively. Prenatal BPA exposure induced the enhancement of both RhoA and HRas membrane translocation, which is in full agreement with the increased HMGCR protein expression (Figure 6).

**Figure 7:**
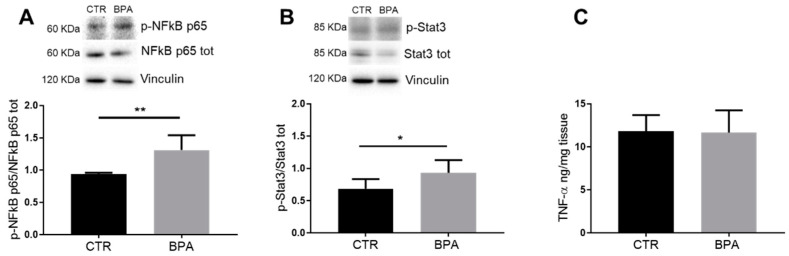
Effects of prenatal exposition to 2.5 µg/kg/day of BPA on inflammatory parameters in fetal livers of female rats. The figure shows typical Western blot of p-NFĸB p65 (**A**) and p-Stat3 (**B**) and their total levels in fetal livers from female CTR animals and the ones prenatally exposed to BPA. The graphs represent the activation state of the proteins in fetal liver lysates. (**C**) Total level of TNF-α in liver lysates of female fetuses measured by ELISA assay kit. Values are expressed as mean ± SD, *n* = 6 for each experimental group. Differences between groups were considered as significant at *p* < 0.05 and were analyzed with unpaired Student’s *t* test. * *p* < 0.5; ** *p* < 0.01 vs. CTR.

Previous work demonstrated that RhoA signaling is involved in inflammation processes activating the RhoA/nuclear factor (NF)κB p65 pathway [39], and, as already reported, BPA caused the activation of inflammatory response [23]. Therefore, we wondered whether prenatal exposure to 2.5 µg/kg/day of BPA alters the level of inflammatory markers in liver of female fetuses. To this aim, firstly, the NFĸB p65 activation state was measured. The results showed that BPA exposure significantly increased the phosphorylation, and therefore activation, of NFĸB p65 (Figure 7A). Successively, we analyzed Stat3, a protein downstream from NFĸB p65 activation, showing it was activated as well (Figure 7B). Conversely, we did not find significant differences in tumor necrosis factor α (TNF-α) expression between BPA-exposed fetuses and control groups (Figure 7C).

## 4. Discussion

The role of BPA in several metabolic pathologies has been highlighted by many lines of evidence, although the network of action is still unsolved and represents a challenge to unveil the underlying molecular mechanisms that can lead to disease onset. The effects of BPA on animals and human health are strictly dependent on dose, onset, and duration of exposure [31]. Importantly, the associated danger increases if exposure occurs during fetal and neonatal life, which are critical developmental windows [40,41]. In this context, the vulnerability to low doses of BPA exposure is still unclear and deserves further studies since it represents a hidden danger. Herein, we demonstrate early and significant effects in the liver following BPA prenatal exposure at a dose almost two times less than the approved human TDI of 4 μg/kg of body weight/day [28,30], in line with data that we already reported [16]. After chronic exposure to 2.5 µg/kg/day of BPA during pregnancy, via drinking water, we did not detect significant amounts of free BPA in the maternal serum, and we did not record variations in serum levels of T-C, TG, LDL, and HDL in response to pollutant exposure. However, additional studies are needed to ascertain whether modulations present in liver of fetuses are consequential to changes in maternal physiology that we did not evaluate in this study, or to the direct body burden of BPA in fetuses. In this regard, it has been recently demonstrated in rats that exposing dams to 2.5 µg/kg/day of BPA causes fetal weight gain and increases the ratio between fetuses and placenta weights, meaning a greater efficiency of placenta in response to this chemical [42]. It is important to note that both BPA and its glucuronide can easily pass this placental barrier reaching the fetuses. Moreover, the limited functionality of fetal UDP glucuronosyltransferase, which metabolizes xenobiotics [43], permits a remarkable fetal exposure to BPA even if the maternal one is very low. This fact is supported by the increased levels of ERα that we found in livers of both male and female BPA-exposed fetuses. Indeed, it is well known that this EDC can enhance the levels of this hormone receptor [31,44]. Fascinatingly, BPA exposure increases ERα levels in females making the content the same as those of males. At the used dose, BPA modulates the main proteins that regulate lipid metabolism and inflammation in the livers of fetuses, occurring in a sex-dependent manner. Our results are in line with data reported by other researchers, which indicate that males and females exhibit differential susceptibility to BPA exposure [45,46]. A wide range of physiological factors could be responsible for the differences shown between sexes, which enclose hormonal differences, innate differences in hepatic lipid metabolism, xenobiotic clearance between males and females, and sex-specific placental responses to external cues [47,48,49,50]. Specifically, in our study, prenatal exposure to BPA is linked to female-specific increase in HMGCR and decrease in ACC levels, which are key enzymes in the cholesterol and fatty acid biosynthesis pathways, respectively. The effects of BPA on HMGCR and ACC seem to depend on different mechanisms. Particularly, our results suggest that BPA decreases ACC protein levels through transcriptional mechanisms, according to the decreased expression of nuclear fragment of SREBP1, which is the active form. These results contrast previous studies demonstrating that prenatal exposure to BPA trough drinking water showed enhanced hepatic transcription of *Acc* and *Srebp1c* genes in female rats [23], although this difference may be due to different doses or ages of the experimental models used; moreover, gene transcription does not always mean protein expression. It has been demonstrated that maternal exposure to low doses of BPA during gestation and lactation produces life-long dimorphic modulations in metabolic homeostasis in rat offspring [51,52].

Changes induced by BPA on cholesterol metabolism could be mediated by post-translational regulatory mechanisms. Indeed, the active fragment of transcription factor SREBP2 showed unaltered levels in the treated group, while the greater BPA-induced amount of the HMGCR protein level seems to rely on the reduction in the HMGCR degradation rate. The hepatic levels of LDLR, another major target of nSREBP2, showed no modulations in animals exposed to BPA, while the observed female-dependent modulation of other lipoprotein receptors and transporters (LRP1, SR-B1, and ABC1) may depend on a homeostatic response to keep the cellular lipid content constant. In fact, despite the significant modulations of HMGCR and ACC, the tissue content of cholesterol and triglycerides was not altered in female fetuses exposed to BPA compared to controls. Interestingly, the absence of unesterified cholesterol content, both in controls and in exposed female fetuses, indicates that at this developmental stage, the liver does not store cholesterol, and that BPA does not induce cellular accumulation of this lipid.

Besides cholesterol, HMGCR takes part in the synthesis of other important end-products, named prenyls. In turn, changes in the availability of these compounds can affect the protein prenylation state [16,38]. The covalent attachment of prenyls, such as farnesol and geranylgeraniol, assures membrane anchoring, and thus the activation of the small GTPases HRas and RhoA, respectively. The obtained data indicate that prenatal BPA exposure promotes RhoA and HRas translocation to membranes only in female rat fetuses, in agreement with the increased HMGCR levels. These small GTPases proteins are involved in a plethora of signal transduction pathways, primarily regulating cytoskeletal dynamics, and affecting many cellular processes, e.g., cell polarity and migration, vesicle trafficking and cytokinesis [53]. Nevertheless, recent studies sustained the involvement of HRas in energy homeostasis. For instance, it is reported that the HRas/ERK pathway plays a relevant role in the signaling network that regulates hepatic metabolism in response to insulin [54]. Studies carried out in transgenic mice highlighted the causative correlation between a reduced levels of HRas and the onset of diet-induced obesity, insulin resistance, and liver steatosis resistance [55,56]. Therefore, alterations in these key proteins could disrupt the proper function of the liver and enhance the risk for metabolic disorders in adolescence and adulthood. Moreover, numerous studies reveal that Rho family members contribute to pro-inflammatory processes [57,58]. As mentioned above, it has been demonstrated that RhoA signaling is involved in inflammation processes activating the RhoA/NFkB p65/Stat3 pathway [39]. Moreover, several lines of investigation suggest that the inhibition of protein geranylgeranylation could be a strategy to cure inflammatory diseases. In this context, statins, which are irreversible inhibitors of HMGCR, show also a powerful anti-inflammatory effect counteracting the upregulation of pro-inflammatory molecules such as interleukin (IL) 1β, IL-6, and tumor necrosis factor alpha (TNF-α) through the repression of prenylation [58,59,60]. Therefore, the activation of RhoA together with NFĸB P65 and Stat3, two key transcriptional factors involved in inflammation [61,62] observed in our study, provided evidence that activation of inflammatory pathways occurs early in life after exposure to very low doses of BPA, and that this alteration may occur through the modulation of HMGCR. However, the absence of BPA-induced modulations in TNF-α is not in agreement with previous findings showing enhanced levels of pro-inflammatory cytokines, such as IL-6 and TNF-α, upon BPA treatment [23,63]. Once again this can depend on the different ages, doses, routes of exposition, and experimental models used. Despite that BPA exposure is associated with activation of the inflammatory response, the mechanisms are still not completely understood. It has been hypothesized that BPA can also trigger Toll-like receptors (TLRs), which in turn may induce Jun N-terminal kinase (JNK) and NFĸB pathways, leading to up-regulation of pro-inflammatory factors [64].

Overall, our findings further strengthen the evidence for sex-specific effects of BPA, suggesting that exposure to a very low dose of this chemical during prenatal life induces metabolic alterations in early stages of development. Despite that we examined only fetuses, we hypothesize that the observed modulations may contribute to the onset of metabolic diseases late in life. Indeed, the concept of “developmental programming” strongly supports that stimuli occurring during the intrauterine life can alter the metabolic pathway and program hepatic development and function [65,66], and these stimuli, including BPA exposure, could then govern the response to postnatal lifestyle factors, such as diet. However, future studies are critical to address this topic and to analyze the mechanisms that determine the prominent sex differences observed in the hepatic response to BPA. Moreover, we can also speculate that the observed effects could be even worse in humans. In fact, pregnancy is longer in humans than in rodents, and fetal vessels in humans are separated from the mother blood by only one layer of syncytiotrophoblast, while in rodents there are two layers. This means that human fetuses are exposed to BPA longer and are less protected [67].

## Figures and Tables

**Table 1:** Serum free BPA level and lipid levels of dams. Dams received 0.05% ethanol (control group, CTR) and 2.5 µg/kg/day of bisphenol A (BPA) for one month before and during pregnancy. Values are expressed as mean ± SD, *n* = 6 for each experimental group. Differences between groups were considered as significant at *p* < 0.05 and were analyzed by unpaired Student’s *t* test.

Groups	Free BPA (pg/mL)	TC (mg/dL)	TG (mg/dL)	HDL-C (mg/dL)	LDL-C (mg/dL)
CTR	12.61 ± 0.92	88.00 ± 14.44	299.67 ± 104.86	61.33 ± 10.89	16.50 ± 9.42
BPA	13.98 ± 3.44	70.80 ± 17.41	366.20 ± 142.72	46.00 ± 12.21	10.00 ± 2.55

TC: total cholesterol; TG: triglycerides; HDL-C: high density lipoprotein- cholesterol; LDL-C: low density lipoprotein- cholesterol.

**Table 2:** Effects of prenatal exposition to 2.5 µg/kg/day of BPA on hepatic cholesterol and triglycerides content in female fetus rats. Total cholesterol, free cholesterol, and triglycerides were measure in liver of female fetuses (CTR F = female control; BPA F = female exposed to BPA) *n* = 6 for each experimental group. Differences between groups were considered as significant at *p* < 0.05 and were analyzed with unpaired Student’s *t* test.

Lipid Content	CTR F	BPA F
Total cholesterol (µg/mg tissue)	2.78 ± 0.41	2.95 ± 1.01
Free cholesterol (µg/mg tissue)	2.51 ± 0.51	2.70 ± 0.75
Triglycerides (nmol/mg tissue)	2.03 ± 0.27	1.92 ± 0.16

## Data Availability

The data presented in this study are available on request from the corresponding author.
